# Supplementary material for: Bioinformatics Identification of Regulatory Genes and Mechanism Related to Hypoxia-Induced PD-L1 Inhibitor Resistance in Hepatocellular Carcinoma
Source: Int J Mol Sci. 2023 May 13;24(10):8720. doi: 10.3390/ijms24108720 (PMC10218698; doi:10.3390/ijms24108720)
Supplement: Supplementary file 1 [file ijms-24-08720-s001.zip › ijms-2293321-supplementary.pdf]

Table S1. RNAseq GEO Signatures Human enrichment analysis of HSGs and HRGs.

	Gene set	Overlap	Adjusted *P*-value	Genes	Databases
HSGs	Rb Immunity Downregulating Pd-L1 GSE109724 1	12/250	0.022	TPX2*; KIF20A*; CENPA*; DLGAP5*; LMNB1*; HILPDA; TM4SF1; GNAZ; CDK1; HMGB2; KDELR3; APOBEC3B	RNAseq_Automatic_GEO_Signatures_Human_Down
HSGs	Rb Immunity Downregulating Pd-L1 GSE109724 3	12/250	0.022	LMNB1*; HILPDA; TM4SF1; ZNF143; SERPINB1; CDCA4; PDGFA; SFN; EPPK1; SOX12; DKK1; SOX4	RNAseq_Automatic_GEO_Signatures_Human_Down
HRGs	Rb Immunity Downregulating Pd-L1 GSE109724 1	16/250	3.982×10^{-4}	CENPA*; LMNB1*; TPX2*; KIF20A*; DLGAP5*; KIFC1; TNFAIP8L1; RAD54L; CDCA3; DHCR24; PSRC1; PBK; NUSAP1; FAM64A; KIF2C; FAM72D	RNAseq_Automatic_GEO_Signatures_Human_Down
HRGs	Rb Immunity Downregulating Pd-L1 GSE109724 3	14/250	0.009	PRDM8; DUSP5; SEMA4B; PDGFB; NDRG1; VEGFA; FOSL1; ABTB1; GPRC5A; PLCH2; ARHGEF2; ITGA5; RIN2; EPHA2	RNAseq_Automatic_GEO_Signatures_Human_Down
HRGs	Tissue-Resident Pancreas Pd-1 Pd-L1 GSE135582 3	29/250	2.008×10^{-12}	PPP1R15A; USP36; CSRNP2; ANKRD37; PFKFB3; METRNL; HERPUD1; RND1; ZFP36; RASD1; SERTAD1; NAMPT; PMAIP1; DNAJB9; PIM3; TSPYL2; EGR1; DUSP5; GABARAPL1; WSB1; FOS; TUBA4A; FOSL2; VEGFA; PER1; DDIT3; MAFF; JMY; FOSB	RNAseq_Automatic_GEO_Signatures_Human_Up
HRGs	Tissue-Resident Pancreas Pd-1 Pd-L1 GSE135582 1	14/250	0.004	TSPYL2; USP36; GABARAPL1; PFKFB3; ARID5A; FOS; METRNL; NDRG1; FOSL2; PER1; ZFP36; NAMPT; IDS; PMAIP1	RNAseq_Automatic_GEO_Signatures_Human_Up
HRGs	Rb Immunity Downregulating Pd-L1 GSE109724 1	12/250	0.023	TSPYL2; ARRDC4; KLF10; PPP1R15A; EGR1; PER1; USP36; DUSP1; MAFF; FOSB; FOS; ALOXE3	RNAseq_Automatic_GEO_Signatures_Human_Up

[1] *Represents duplicate genes in HSGs and HRGs

Table S2. The multiple regression model identified 14 effector genes affecting the PD-L1 expression: Coefficient associated with the expression level of effector gene, the corresponding t-score and *p*-value.

Effector gene	Co-efficient, b	t	*p*
const	0.076	1.897	0.059
DLGAP5	0.443*	3.6	3.636×10^{-4}
NDC80	0.41*	3.292	0.001
LMNB1	0.317*	3.062	0.002
FAM13A	0.261	5.893	8.770×10^{-9}
FOS	0.24	5.369	1.434×10^{-7}
GABARAPL1	0.23	4.595	6.004×10^{-6}
PIK3R1	0.145	2.57	0.011
EPHA2	0.121	2.593	0.01
NEDD4L	-0.096	-2.008	0.045
CABYR	-0.123	-2.971	0.003
HGFAC	-0.15	-3.599	3.652×10^{-4}
ALDH5A1	-0.264	-5.006	8.740×10^{-7}
KIF20A	-0.418*	-3.472	5.803×10^{-4}
TPX2	-0.434*	-3.156	0.002

[1] *Represents PD-L1 regulator genes with the top five coefficient magnitudes

Table S3. Kaplan-Meier survival analysis and ROC of hub genes and PD-L1 regulator genes of hepatocellular carcinoma

Gene name	Hub gene	Regression	OS PD-L1	RFS	PFS	DSS	Response to PD-L1 inhibitor
CCNB1	√		0.3	1.2×10^{-4}*	6.9×10^{-6}*	5.3×10^{-4}*	0.3
BUB1B	√		0.33	0.014*	1.1×10^{-4}*	1.2×10^{-4}*	0.33
KIF4A	√		0.85	0.005*	2.6×10^{-4}*	1.7×10^{-4}*	0.37
KIF11	√		0.51	6.8×10^{-4}*	2.6×10^{-5}*	4.0×10^{-4}*	0.24
CENPA*	√		0.094	0.009*	0.001*	4.1×10^{-4}*	0.22
POLE2*	√		0.081	0.77	0.087	0.029*	0.027*
KIF20A*	√	√	0.14	2.7×10^{-4}*	1.8×10^{-5}*	1.6×10^{-4}*	0.22
NDC80*	√	√	0.024*	0.017*	0.003	0.004*	0.076
TPX2*	√	√	0.03*	0.001*	6.6×10^{-6}*	6.8×10^{-6}*	0.28
DLGAP5*	√	√	0.54	0.006*	1.0×10^{-4}*	1.4×10^{-4}*	0.38
FOS		√	0.2	0.47	0.67	0.71	0.26
FAM13A		√	0.92	0.8	0.87	0.77	0.08
ALDH5A1		√	0.35	0.13	0.033*	0.023*	0.31
GABARAPL1*		√	0.096	0.23	0.007*	0.002*	0.016*
CABYR		√	0.17	0.07	0.089	0.1	0.15
PIK3R1*		√	0.74	0.05*	0.13	0.007*	0.04*
HGFAC		√	0.52	0.023*	0.026*	0.14	0.26

LMNB1*	√	0.1	0.03*	0.002*	0.018*	0.5
EPHA2	√	0.88	0.12	0.051	0.22	0.12
NEDD4L	√	0.36	0.68	0.91	0.7	0.4

[1] *Represents hub genes and PD-L1 regulator genes with the top five coefficient magnitudes

RFS: Relapse-Free Survival; PFS: Progression-Free Survival; DSS: Disease-Specific Survival

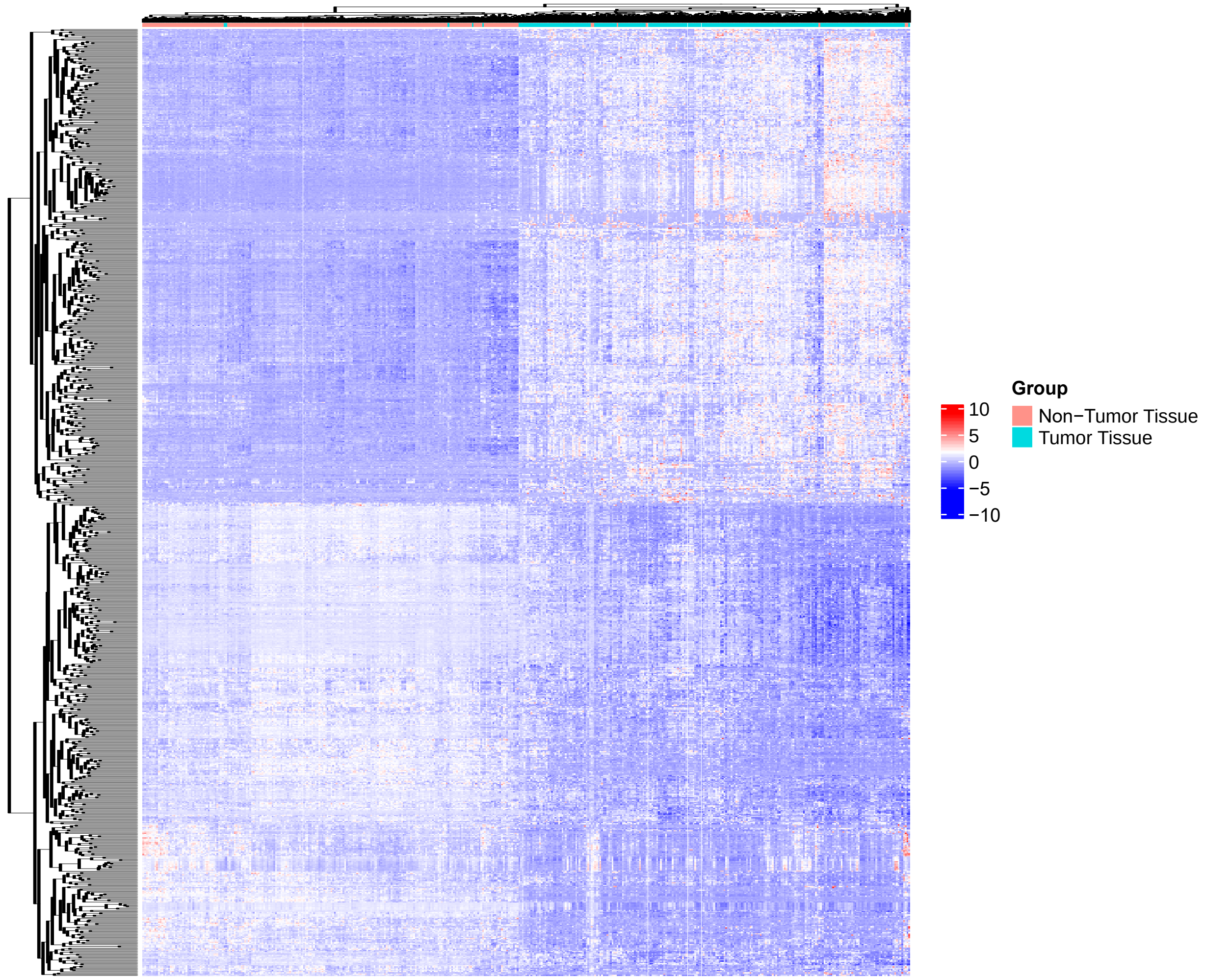

Figure S1 Heatmap for HSGs expression levels

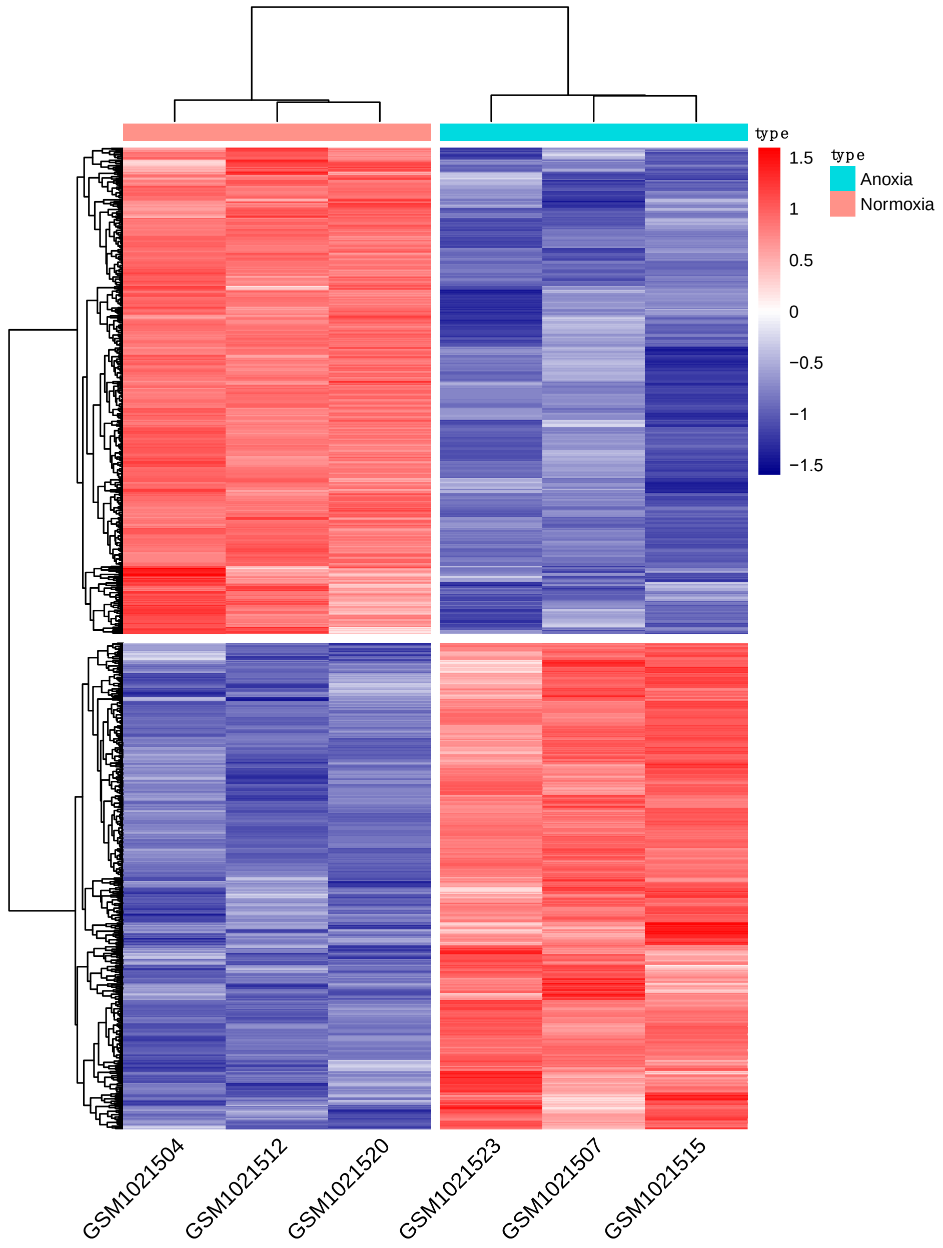

Figure S2 Heatmap for HRGs expression levels

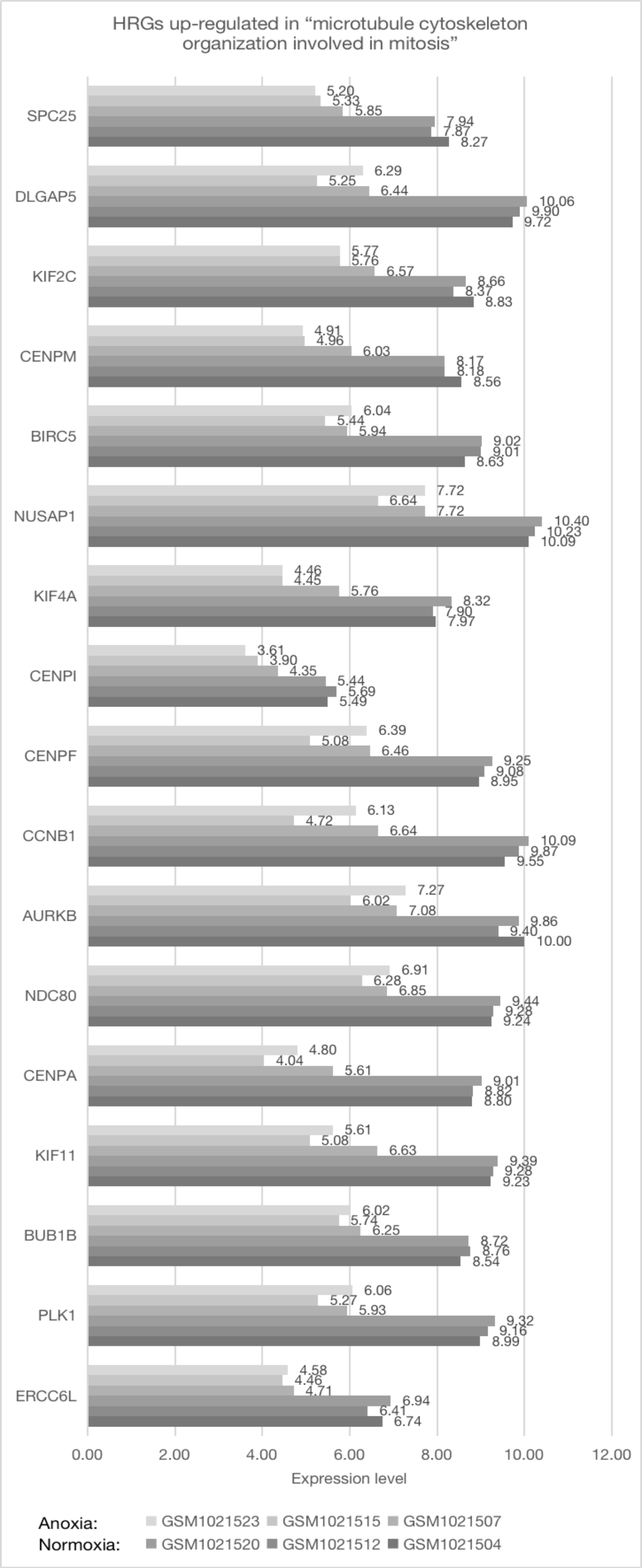

Figure S3 Bar chart of expression profiles of HRGs up-regulated in "microtubule cytoskeleton organization involved in mitosis"
